# DIRECT, a low-cost system for high-speed, low-noise imaging of fluorescent bio-samples

**DOI:** 10.1364/BOE.486507

**Published:** 2023-05-08

**Authors:** Isabell Whiteley, Chenchen Song, Glenn A. Howe, Thomas Knöpfel, Christopher J. Rowlands

**Affiliations:** 1 Department of Bioengineering, Imperial College London, London, UK; 2 Centre for Neurotechnology, Imperial College London, London, UK; 3 Department of Brain Sciences, Imperial College London, London, UK; 4 Department of Physics, Hong Kong Baptist University, Kowloon Tong, Hong Kong

## Abstract

A targeted imaging system has been developed for applications requiring recording from stationary samples at high spatiotemporal resolutions. It works by illuminating regions of interest in rapid sequence, and recording the signal from the whole field of view onto a single photodetector. It can be implemented at low cost on an existing microscope without compromising existing functionality. The system is characterized in terms of speed, spatial resolution, and tissue penetration depth, before being used to record individual action potentials from ASAP-3 expressing neurons in an *ex vivo* mouse brain slice preparation.

## 1. Introduction

There are many advantages to using optical microscopy to investigate biological systems such as cells, tissues and optically accessible organs. It can be fast, can resolve single cells and sub-surface features, and causes little damage to the sample under investigation [1]. Nevertheless, cutting-edge biological applications have ever-more-stringent requirements in terms of speed, limits on photobleaching and tolerance of tissue scattering, all while maintaining the spatial resolution and field of view (FOV) that researchers have come to expect from their microscopes.

Achieving these performance improvements in the general case (for which the structure of the sample is unknown) is an enormous engineering challenge, but in a subset of applications, what matters is not the structure itself, but how its optical properties change over time. This is particularly true in the case of fluorescent sensors, in which probed cells do not move on the timescale of the experiment, but fluorescence emission wavelength, intensity or fluorescence lifetime *does* change. For example, neuroscientists increasingly use optical techniques to monitor calcium ion concentrations and membrane potentials in populations of neurons, by genetically-expressing a fluorescent probe and monitoring the resulting signal using a microscope. Many biological fields use Förster resonance energy transfer (FRET) sensors that can respond very rapidly to external conditions [2]; they are widely used to monitor protein interactions and conformational changes [3], metal ions [4], metabolites and biomarkers [5] and for fluorescence lifetime imaging [6] in live *in vivo* samples. As many of these applications require long-term monitoring, they require optical techniques that can minimize photobleaching, improve temporal resolution (which can be as high as ∼1kHz in the case of some voltage sensors [7]) and reduce the impact of tissue scattering.

So high is the demand for high-speed, targeted systems that a number of techniques have recently been developed that are able to optically target features such as individual neurons within a labelled population, while also achieving improved spatial and/or temporal resolutions. Various multi-beam scanning methods have been developed, [8–10] using galvanometric (galvo) scanning mirrors to move laser foci across a sample, and then recording onto a camera. Multiphoton excitation is commonly used in these applications, to increase tissue penetration while reducing photodamage, but these setups are limited in speed due to their mechanical galvo mirrors, limited camera frame-rates or requirements for pixel binning. Some do not describe the rate of imaging, rather they optimize the spatial resolution of the system [8], others use biological sensors that do not require high speed imaging, so image at low rates of 30 Hz [9], and while the final cited paper does achieve 1kHz camera recording, it is at the expense of the field of view of the camera [10]. Other methods, such as two-photon FACED microscopy, also split the laser into beamlets (this time distributed into a tilted line), recording the fluorescence signal onto a photomultiplier tube sampling at hundreds of megasamples per second [11,12]. Nevertheless, the frame rate is still limited by the need to scan the line using a galvo mirror, requiring a trade-off between temporal and spatial resolutions, as well as being a highly complex and expensive optical system to implement.

Liquid crystal spatial light modulators (lcSLMs) combined with computer generated holographic illumination (CGHI) have been used in targeting systems, primarily in optogenetic targeting, where neurons are targeted simultaneously and recording rates were not critical [13,14]. They have also been used in the activation of neurons, where a camera recorded at high frame rates (>6000 frames per second), however this caused distortion in the image that required multi trial averaging to remove [15]. These examples are limited in the number of targets that can be addressed simultaneously, and do not achieve the speeds desirable for distortion-free imaging of fluorescent voltage sensors and other high-speed probes. Two-photon fluorescence excitation with temporal focusing on the other hand can be used to precisely target thousands of individual neurons at sampling rates up to 160 Hz [16]. Though this approach enables spatially-precise lateral and axial resolution, the limited temporal resolution of the highlighted system precludes its use in optical targeting experiments where there are rapid fluctuations in fluorescence in a densely-populated or scattering sample, and furthermore, as the number of targets is increased, the temporal resolution must be further decreased. Though high-speed multiphoton targeting has been applied over large volumes, the lasers required are high-maintenance and expensive, thus the use of single-photon excitation would be advantageous.

Spatially precise targeting has also been achieved in neurons with a digital micromirror device (DMD) for both optogenetic activation [17–19] and imaging with genetically encoded voltage indicators (GEVIs) [20]. A DMD consists of an array of micromirrors that can take one of two orientations (turning a pixel on or off in effect) at frame rates on the order of tens of kilohertz. In all cases – activation and imaging of neurons – the DMD provided precise spatial resolution and reduced photobleaching, but its high speeds were not utilized to increase temporal resolution; a single frame was projected by the DMD to illuminate all targets, rather than switching between targets at high speed. Neurons were recorded from at a frame rate of 500 Hz, however this recording rate required a reduction of the camera’s FOV through pixel binning to achieve the frame rates fast enough to record high-speed fluorescence changes in GEVIs. Using a camera to record the activity of the targeted regions is the spatially- and temporally-limiting factor in GEVI imaging experiments; researchers are required to optimize the setup towards either the field of view of the camera or to the temporal resolution of the system.

DMDs have also been used to temporally pattern targeted beamlets and measure fluorescence activity onto a PMT. A SLM was used to split the light into beamlets and a DMD was used to project the beamlets in different temporal patterns for each targeted region so that the PMT recording could be separated into individual traces for each targeted region. The high switching rates of the DMD were utilized to generate unique temporal patterns for each targeted region, however they were only able to shape the beamlets into single or small numbers of points rather than customizing the shape to the target image. The authors additionally highlight that imaging speed of scanless imaging methods are limited by camera frame rates, and as such they use a PMT to collect the photon of their system. The effect of light scattering on spatial discrimination is an additional problem highlighted in their paper [21]. This optical system is capable of targeting and recording from multiple regions of a sample simultaneously, however it is a highly complex setup and has not been optimized for recording above 90 Hz.

Given the above techniques, there is a clear need for a low-cost method that can record changes in fluorescence with high sensitivity and ∼500-1000 Hz bandwidth, while ideally preserving the favorable photobleaching characteristics and tolerance to tissue scattering that existing methods provide, compared to simple wide-field microscopy. To address this need, we present *DMD Imaging with Rapid Excitation and Confined Targeting* (DIRECT), a method for fast recording of fluctuating fluorescence signals based on strong structural priors. It uses a DMD to project patterns onto the sample in rapid sequence, recording the fluorescence emission from each projected pattern using a single point detector. The high speed of the DMD theoretically permits more than a dozen regions of interest (ROIs) to be probed with speeds of over 1000 measurements per second, preserving the field of view of the system, while maintaining precise spatial resolution and allowing high-bandwidth recording of fluorescence changes with minimal crosstalk between ROIs. DIRECT exhibits minimal photobleaching, significantly higher speeds, reduced read noise, has less onerous requirements for high-speed data storage, and increased tolerance to tissue scattering relative to conventional wide-field imaging or DMD targeting using a camera, while remaining low-cost, easy to implement and compatible with almost any microscope.

## 2. Results and discussion

### 2.1 Design

**Fig. 1.**
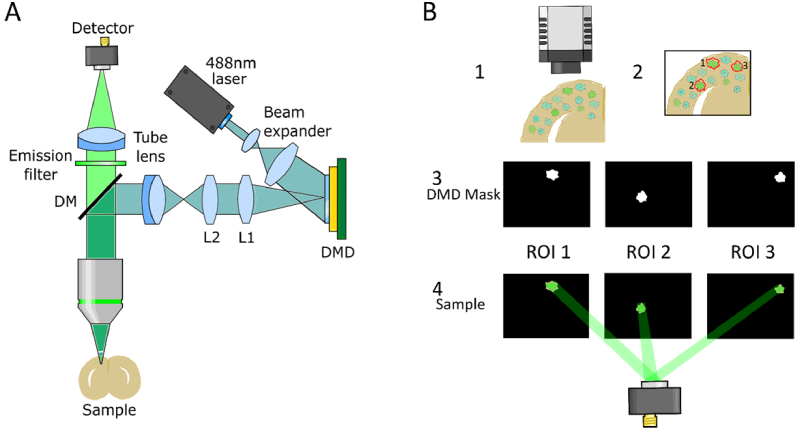
A. Imaging pathway for targeted illumination. Light from a 488 nm laser passes through a beam expander before striking a DMD. The reflected light is projected through a 4f lens system (L1 and L2) and enters the microscope through a tube lens. The light reflects off a dichroic mirror (DM) and through the objective onto the sample. The emitted light returns through the objective and DM, then passes through an emission filter and tube lens. The light is recorded by a detector (PMT, photodiode, SiPM, or camera). A removable cube containing a prism mirror is located after the beam expander to permit illumination with an LED if desired. B. Experimental schematic. 1. Camera takes a picture of a widefield image of sample. 2. Regions of interest are drawn around selected neurons. 3. Masks of targeted neurons are sequentially displayed by the DMD. 4. DMD masks are imaged and emitted light is captured by a PMT.

DIRECT (Fig. 1) was designed to be integrated into standard upright microscopes using widely available off-the-shelf parts and with minimal disruption to the existing microscope. It integrates into the infinity path of the microscope, between the objective and the tube lens. A custom-machined part couples DIRECT to the microscope; it incorporates Olympus microscope dovetails with a Thorlabs removable filter cube holder but can be easily adapted to dovetails from other microscope manufacturers.

A DMD is placed conjugate to the sample plane. Binary masks of the desired targets are projected onto the sample in quick succession (up to 22kHz frame rate) by the DMD. DMDs provide advantages over other light shaping devices such as lcSLMs due to their fast refresh rates (allowing for many target regions to be imaged sequentially) and relatively low cost. After the sample is excited, the resulting fluorescence photons are captured by a single-point detector, such as a photodiode, photomultiplier tube (PMT), or silicon photomultiplier (SiPM); a camera is also available for wide-field imaging. A theoretical comparison of detectors is given below.

It is worth noting that while DIRECT was designed to be used with a single photon laser as a low cost, alternative method to two-photon scanning techniques, the concept can be extended to widefield multiphoton excitation. To do so, a widefield two-photon method, such as temporal focusing, must also be implemented. This comes at the cost of complexity and expense without necessarily providing significant performance advantages. With a single-photon laser, DIRECT can provide precise spatial resolution and multi-depth targeting without requiring a complex two-photon optical setup.

### 2.2 Detector comparison

DIRECT can use many different detectors, including photodiodes, PMTs, SiPMs (also known as Multi-Pixel Photon Counters), avalanche photodiodes and so on. Several of these were available in the laboratory, and their dynamic range, photon detection efficiency and maximum rate of photon detection were compared to establish their utility under different imaging conditions (details in Supp. S1).

Neurobiological experiments in particular have exacting requirements; a very small change in fluorescence must be recorded at high speed (∼1% change in signal intensity at 1 ms per frame [22]). Because fluorescence signals are ultimately shot-noise limited, the probability distribution of the detected signal is given by a Poisson distribution, the signal-to-noise ratio (SNR) of which can be approximated by $\sqrt{m}$ (where *m* is the number of detected events; photons in this case). Thus the detector must capture at least 10,000 photons to achieve a SNR of 1:1 for a change in fluorescence (ΔF/F) of 1%, but greater values are preferable [23,24].

**Fig. 2.**
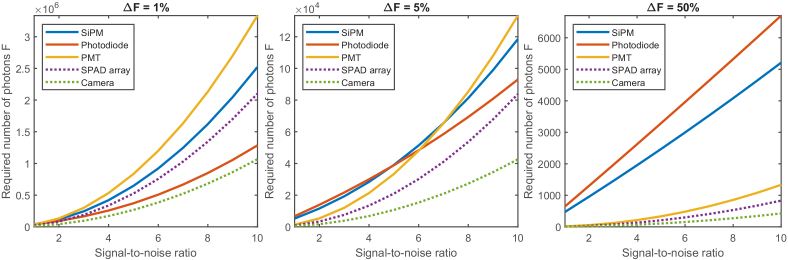
Performance comparison between detectors under different imaging conditions. Simulated frame rate is 1000 frames per second (fps), and there are 10 independent illuminated areas. For cases where the fractional change in fluorescence is small, photon shot noise dominates and detector performance is dominated by the photon detection efficiency. Where the fractional change is large however, detector read noise becomes the dominant effect and low-noise detectors perform better. Note also that the number of photons required to overcome photon shot noise decreases as the fractional change in fluorescence increases, as expected.

The required photon counts for each detector are compared in Fig. 2; all cases describe a system in which integrated photon counts from 10 different areas must be recorded at 1000 frames per second. A detailed description of each detector can be found in Supp. Table S1.

For cases where the fractional change in fluorescence $\Delta F_{\%}$ is low (∼1%, consistent with early voltage sensors like VSFP2s [25] VSFP3s [26], and ArcLight [27]), the dominant factor discriminating between the detectors is the photon detection efficiency. When $\Delta F_{\%}$ reaches ∼5%, the noise of the detectors starts to become important and the PMT starts to become the detector of choice. Once $\Delta F_{\%}$ reaches 50% (consistent with genetically-encoded calcium indicators like jGCaMP7 [28]), the PMT has a clear performance advantage.

For completeness we have also included performance curves for a high-performance camera and Single Photon Avalanche Diode (SPAD) array. For simplicity all simulations assumed 100% of photons emitted by the sample reached the detector and therefore the only reason a photon wouldn’t be detected is due to the detector’s photon detection efficiency, but for reasons detailed in Supp. S1 this simplifying assumption should not be relied upon; care must therefore be taken when comparing these two detectors with the PMT, photodiode and SiPM.

### 2.3 Resolution and scattering tolerance

**Fig. 3.**
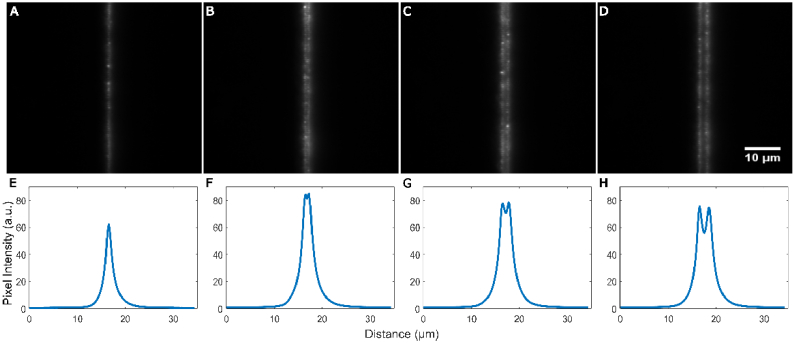
Spatial resolution of DIRECT’s targeting, A. a single pixel width line, B. a double pixel width line, C. two single pixel width lines with a line of off pixels in between, D. two single pixel width lines with two lines of off pixels in between. E-H. average intensity plots of the above lines; the mean of all the pixel values in each column are plotted to ascertain whether there is a reduction in intensity between the projected image of the mirrors

Because the system was optimized such that the DMD image substantially covered the FOV (rather than maximizing spatial resolution over a smaller area), the achievable resolution was limited by the size projected image of the DMD mirrors. This was first tested under ideal, non-scattering conditions. To test whether the relay optics were sufficient to maintain the resolution set by the DMD pixel size, a thin sample consisting of a monolayer of 100 nm fluorescent beads was prepared. Single-mirror-width lines were projected onto the sample; images were captured of a single line, two adjacent lines, two lines with a row of off-mirrors in between them, and finally two lines with two rows of off-mirrors in between them (Fig. 3 A-D). The Sparrow limit was selected as the resolution criterion as it is more stringent than the more common Rayleigh criterion [29] while being simple and unambiguous to assess. The Sparrow limit is reached when the dip in light intensity between two points can no longer be resolved; it assumes uniform light intensity for both points. In the targeting resolution experiments, the two projected lines adjacent to each other, with no off-mirrors in between are resolvable by the Sparrow criterion as the two separate intensity peaks are clearly visible (Fig. 3(B),(F)). The resolved gap is the gap between the mirrors of the DMD (i.e. at least 13.7 µm/20 = 685 nm or better), thus we can conclude that the resolution of DIRECT is only limited by the mechanical design of the DMD.

The average pixel intensity of the projected lines was measured (Fig. 3 E-H); the full-width, half-maximum (FWHM) of the plots was acquired and then compared to the theoretical FWHMs of a diffraction limited system. For a single row of mirrors, the FWHM was 1.69 µm and the theoretical was 0.71 µm at the sample plane. The acquired FWHMs for the other instances were 2.60 µm, 3.23 µm, and 3.80 µm respectively. For the different images we found the FWHM of the acquired data to be on average 1.04 µm ± 0.094 µm wider than the theoretical FWHMs. We believe that much of the increase in width is due to light scattering from the beads.

**Fig. 4.**
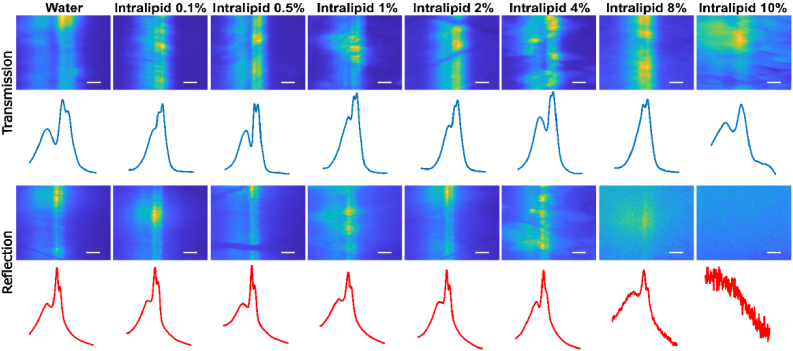
Transmission and epifluorescence images of two single mirror width lines, separated by two columns of off mirrors (a separation of 2.1 µm in the sample plane), projected through a scattering phantom. Dilutions of intralipid 20% were used to increase scattering density. Plots show the average pixel intensity of the projected lines. Scale bar = 5 µm

DIRECT was designed to be used for biological tissues and therefore needs to be able to target ROIs through dense scattering. Because all scattered photons are integrated onto a single detector (thus scattering of emission photons has negligible effect), DIRECT is expected to have heightened tolerance to tissue scattering compared to techniques like confocal microscopy. Nevertheless, because the optical properties of tissue samples are difficult to control, due to reasons such as location of targeted region, age of sample, and differences between samples, scattering phantoms (with controllable levels of light scattering) were used as a proxy. As in the above targeting resolution experiment, lines were projected by the DMD with a separation of 2.1 µm at the sample, and imaged onto a thin layer of fluorescent microspheres. Above the fluorescent spheres, dilutions of Intralipid 20% were used as phantoms to represent different levels of scattering [30] (Fig. 4). For reference, 10% Intralipid has a reduced scattering coefficient $\mu_{s}^{\prime }$ of approximately 80cm^-1^ at 500* *nm [31], roughly twice that of skin, more than three times that of brain tissue, and nearly five times that of breast tissue [32]. The upright microscope’s existing epifluorescence camera was supplemented by a second camera in a transmission geometry. Briefly, a custom-machined adaptor replaced the condenser, allowing an objective to be placed (in a geometry reminiscent of an inverted microscope) under the sample, imaging the fluorescent microspheres. An elliptical mirror in the adaptor enabled the light from the *“*inverted” objective to be projected through a tube lens onto a camera (Fig. S3). Images were taken from both epifluorescence and inverted cameras simultaneously, and their intensities were compared (Fig. 4). With low levels of scattering, projected lines in both the transmission and epifluorescence images were resolvable. As levels of scattering increased, the transmission images could still be resolved, however the epifluorescence images were not resolvable. In densely scattering situations, DIRECT was still able to achieve resolvable images, meaning it can achieve precise spatial targeting, even if the epifluorescence image was not visible to the camera through the scattering. As a result, the photons travelling back through scattering medium are all from the targeted ROIs, and can be collected by single-point detectors such as photodiodes or PMTs as signals from the ROI. Therefore, DIRECT outperforms camera detection of ROI projection by accurately targeting and recording from ROIs even when the fluorescence photons are scattered.

### 2.4 Photobleaching

Photobleaching is a persistent issue in many biological experiments [33]. To reduce it, the excitation power can be reduced, but this can cause increased photon shot noise, reducing the signal-to-noise ratio as noted in section 2.2. Additionally, regions that are not actively being recorded from are still exposed and subject to photobleaching, which is damaging to the sample and can limit experimental utility. Because DIRECT does not illuminate cells that are not being recorded from, it should substantially reduce off-target photobleaching.

**Fig. 5.**
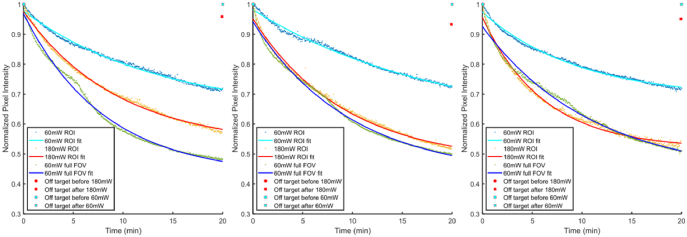
Photobleaching decay curves for 60 mW ROI exposure (cyan curve, blue datapoints, simulating DIRECT), 180 mW ROI exposure (red curve, orange datapoints, simulating DIRECT at higher power) and 60 mW widefield illumination (blue curve, green datapoints), three repeats. Curve fits for 60 mW ROI were ${0.34e}^{-2.4\times 10^{-3}x}+0.64$ , ${0.39e}^{-1.8\times 10^{-3}x}+0.59$ , and ${0.29e}^{-3.4\times 10^{-3}x}+0.69$ respectively, curve fits for 180 mW ROI were ${0.45e}^{-3.5\times 10^{-3}x}+0.53$ , ${0.48e}^{-3.6\times 10^{-3}x}+0.47$ , and ${0.44e}^{-5.4\times 10^{-3}x}+0.52$ respectively, and curve fits for 60 mW widefield illumination were ${0.54e}^{-4.1\times 10^{-3}x}+0.43$ , ${0.51e}^{-3.4\times 10^{-3}x}+0.43$ , and ${0.50e}^{-3.0\times 10^{-3}x}+0.43$ respectively. For the two ROI measurements the photobleaching of all non-targeted regions was also compared; for 60 mW ROI the fluorescence intensity dropped to a mean of 0.9897 of initial values, and for 180 mW ROI intensity dropped to 0.9478

Photobleaching rates for widefield fluorescence and DIRECT were compared. A series of images were captured over 20 minutes using both techniques: uniform illumination in the case of widefield fluorescence imaging, or switching between ROIs in the case of DIRECT. The average pixel value of each captured image or targeted region within the image was calculated and normalized to the exposure time, and then an exponential decay curve with an offset was fitted to each dataset (Fig. 5). At the same laser power and switching between three ROIs (∼ 55 µm diameter circles) at 100 frames per second, DIRECT has a pronounced improvement in rate of decay compared to widefield imaging. When the laser power was increased by a factor of three to account for the reduced amount of time DIRECT spends illuminating each target, we found the decay rate to be similar to, or in some cases better than, the widefield illumination (Fig. 5). To assess off-target photobleaching, full FOV before and after images were captured for the ROI measurements. The off-target after values were normalized to the values in the “before” image and showed minimal reduction of pixel intensity. This highlights that DIRECT improves tolerance to on-target photobleaching and exhibits minimal off-target photobleaching, and provides further support for the benefits of using DIRECT.

### 2.5 Voltage imaging

While DIRECT can be used for many applications, it is particularly suited to the high-speed high-dynamic-range measurements needed for imaging genetically-encoded fluorescent voltage indicators in brain tissue [22]. For this demonstration, DIRECT was used to record from multiple neurons simultaneously in an *ex vivo* mouse brain slice expressing soma-targeted ASAP3 in cortical neurons. ASAP3 is a genetically-encoded voltage sensor which changes fluorescence intensity in response to changes in cell membrane potential [34]. While many genetically-encoded voltage sensors operate in this manner, typically the actual fluctuations are small and they are prone to photobleaching [22]. Therefore, they require low background noise and high-speed imaging to resolve the optical voltage signals. The imaging system must also achieve a high spatial resolution to avoid both sampling from and bleaching of the voltage indicator-expressing neurons that are not of interest. DIRECT is an ideal system for recording from voltage indicators as its high speed can capture the fluctuations in fluorescence, and its targeting reduces background noise and off-target bleaching, while also reducing on-target photobleaching by decreasing the time the light spent on each target. A comparison of DIRECT versus conventional widefield imaging can be seen in Supp. section S5.

**Fig. 6.**
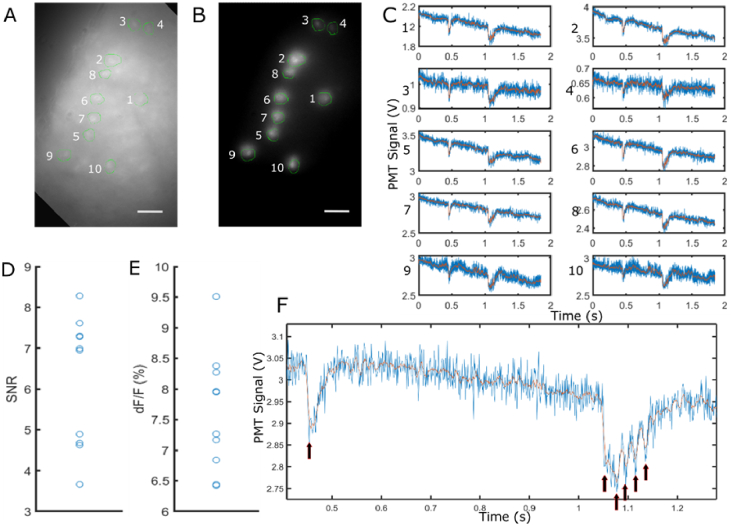
Single trial multi-cell recording. A. Widefield FOV of the sample with selected neurons circled in green, scale bar = 50 µm. B. Targeted illumination of the sample; ROIs for each neuron shown in green, scale bar = 50 µm. C. Single trial traces of each ROI on left during extracellular stimulation experiment. Electrode was placed in proximity to the targeted neurons. A single pulse was given at approximately 0.5s into the experiment, followed by 5 pulses half a second later. Action potentials are visible at each pulse for each neuron. No photobleaching correction has been applied. Note that ASAP3 voltage indicators decrease in fluorescence during an action potential. D,E. Signal to noise ratio and change in fluorescence for the first action potential of each neuron (n = 10). F. Close view example of single trial data in C, black arrows indicate voltage response to each extracellular electrical pulse. Blue is raw data, orange line is smoothed data using a moving average filter with a span of 10 data points.

As a final demonstration, DIRECT was used to record electrical stimulation-induced excitation in ASAP3-expressing neurons in an *ex vivo* slice preparation across an 875 µm × 656 µm FOV. Unlike other neuronal activity indicators, ASAP3 decreases in its level of fluorescence, rather than increasing, when the neuron is depolarizing towards an action potential. Masks of each targeted neuron were generated and projected onto the sample for 100 µs before switching to the next mask. The emitted photons were collected by a PMT. Ten neurons across the FOV were targeted (Fig. 6(A),(B)) and the PMT recorded at 1 MS/s (megasamples per second); the high sampling rate enables accurate off-line synchronization of the measured data. There was an effective sampling rate of 1 kHz per neuron determined by the DMD pattern cycle time. A stimulation electrode was placed into the sample at a close proximity to the targeted neurons and a short electrical pulse was given, followed by 5 consecutive pulses 0.5 sec later. Activity traces of the 10 neurons were separated (Fig. 6(C),(F)) and the ΔF/F and SNR ratio for the first action potential was calculated. The average SNR for the first action potential was 6.23 (σ=1.59, n = 10 neurons) and the mean ΔF/F of ASAP3 during the first action potential was -7.62% (σ=0.0098, n = 10 neurons; negative-going signal reflects depolarization) (Fig. 6(D),(E)). The ΔF/F of was lower in value than other papers [34] have reported likely due to autofluorescence, scattering of the excitation light and increased fluorescence background of the system. The average intensity of each targeted neuron differed due to the different axial depths of each neuron, however the signal to noise ratio was high enough to resolve the changes in fluorescence that occurred due to electrical stimulation, even at the lowest baseline fluorescence values. By utilizing DIRECT, and switching between the different targets, the individual activity traces of each neuron could be easily separated and single action potentials for each target were resolved. Though single photon imaging has limited axial resolution when compared to two-photon imaging, its penetration capabilities allow for a focus below the surface of the sample and for identification of targets at different axial depths. As DIRECT only projects a two-dimensional mask, the axial depth of the target does not need to be known when using DIRECT, just its lateral location at the sample. As seen in Fig. 4, DIRECT is able to accurately illuminate a target even if the optical focus is not in the same axial plane as the target. DIRECT combined with a PMT enabled neurons at different tissue depths to be recorded from simultaneously despite the precise shape of the neuron being unknown.

The total number of ROIs that can be recorded from during a single experiment without losing any activity information is determined by minimum sample rate that can be used, which in turn is determined by the rise and fall times of the fluorescence indicators used. In the above experiments, the system can unambiguously resolve signals with a bandwidth of 500 Hz (Nyquist sampling). If this sampling rate is retained, then DIRECT can target up to 22 ROIs (based on the DMD switching rate).

## 3. Conclusion

In summary, a new targeted imaging system, DIRECT, has been developed to take optical measurements at high frame rates across a wide FOV, without compromising spatial resolution. The system is simple to implement and can be retrofitted with only minor modification to almost any microscope, with minimal disruption to existing functionality; a methods section is in Supp. S6. DIRECT can project patterns as small as a single mirror width of the DMD, allowing for precise targeting resolution. The speed of the DMD allows for target switching as fast as 22 kHz, well above the bandwidth of most fluorescent biological probes; this was demonstrated by recording from ASAP3-expressing neurons in an *ex vivo* acute brain slice preparation.

The system is in active development and routes for further optimization include optimizing system throughput and collection of scattered photons; incorporating detectors with higher quantum efficiency; exploiting the achromaticity of the system to increase temporal bandwidth (by expressing several GEVIs with different fluorescence emission spectra and measuring them simultaneously); and developing a version that integrates the laser, DMD and optics for use by researchers who do not have optical engineering expertise.

## Data Availability

Data, Computer Aided Design (CAD) models and software are available upon request at Ref. [35].

## Supplemental document

See Supplement 1 for supporting content.
