# Supplementary material for: DIRECT, a low-cost system for high-speed, low-noise imaging of fluorescent bio-samples
Source: Biomed Opt Express. 2023 May 8;14(6):2565–75. doi: 10.1364/BOE.486507 (PMC10278627; doi:10.1364/BOE.486507)
Supplement: Supplementary file 1 [file boe-14-6-2565-s001.pdf]

Supplemental Document

Biomedical Optics EXPRESS

DIRECT, a low-cost system for high-speed, low-noise imaging of fluorescent bio-samples: supplement

ISABELL WHITELEY,[1,2,*] CHENCHEN SONG,[3] GLENN A. HOWE,[1] THOMAS KNÖPFEL,[2,3,4] AND CHRISTOPHER J. ROWLANDS[1,2]

[1] *Department of Bioengineering, Imperial College London, London, UK*
[2] *Centre for Neurotechnology, Imperial College London, London, UK*
[3] *Department of Brain Sciences, Imperial College London, London, UK*
[4] *Department of Physics, Hong Kong Baptist University, Kowloon Tong, Hong Kong*
[*] *i.whiteley18@imperial.ac.uk*

This supplement published with Optica Publishing Group on 8 May 2023 by The Authors under the terms of the Creative Commons Attribution 4.0 License in the format provided by the authors and unedited. Further distribution of this work must maintain attribution to the author(s) and the published article's title, journal citation, and DOI.

Supplement DOI: https://doi.org/10.6084/m9.figshare.22591585

Parent Article DOI: https://doi.org/10.1364/BOE.486507

DIRECT, a low-cost system for high-speed, low-noise imaging of fluorescent bio-samples: supplementary information

ISABELL WHITELEY,[1,2,*] CHENCHEN SONG,[3] GLENN A HOWE,[1] THOMAS KNÖPFEL,[2,3,4] AND CHRISTOPHER J ROWLANDS[1,2]

[1]*Department of Bioengineering, Imperial College London, London, UK*
[2]*Centre for Neurotechnology, Imperial College London, London, UK*
[3]*Department of Brain Sciences, Imperial College London, London, UK*
[4]*Department of Physics, Hong Kong Baptist University, Kowloon Tong, Hong Kong.*
*[*i.whiteley18@imperial.ac.uk](mailto:i.whiteley18@imperial.ac.uk)*

S1. Detector Noise Derivation

To compare detectors under various conditions, it is necessary to know the expected photon count per pixel (F) required to overcome the experimental noise. The total noise n_{total} (defined as the mean deviation of a measurement from its true value) can be approximated as the combination of the detector dark noise and the photon shot noise of the overall measurement, summed in quadrature:

$$n_{total} = \sqrt{{n_{shot}}^2 + {n_{dark}}^2}$$

Eq. 1

Since the signal is expressed as a fractional change in fluorescence $\Delta F_{\%}$, the total number of fluorescence photons $F = n_{total}/\Delta F_{\%}$, for a SNR of 1. If the user desires a higher SNR, this expression must be multiplied by the relevant factor. Thus

$$F = SNR\frac{n_{total}}{\Delta F_{\%}}$$

Eq. 2

Finally, because n_{shot} is the standard deviation of the shot noise of the measurement (which can be approximated as $n_{shot} = \sqrt{F}$), the following expression can be obtained:

$$F = SNR\frac{\sqrt{F + {n_{dark}}^2}}{\Delta F_{\%}}$$

Eq. 3

Which can be rearranged into the form of a quadratic:

$$\left(\frac{\Delta F_{\%}}{SNR}\right)^2 F^2 - F - {n_{dark}}^2 = 0$$

Eq. 4

This can be solved using the well-known quadratic formula

$$x = \frac{-b \pm \sqrt{b^2 - 4ac}}{2a}$$

Eq. 5

Substituting $x = F$, $a = (\Delta F_{\%}/SNR)^2$, $b = -1$ and $c = -\,{n_{dark}}^2$, and ignoring the negative solution resulting from subtracting the square root term:

$$F = \frac{1 + \sqrt{1 + 4 \times \left(\frac{\Delta F_{\%}}{SNR}\right)^2 \times {n_{dark}}^2}}{2 \times \left(\frac{\Delta F_{\%}}{SNR}\right)^2}$$

Eq. 6

For comparison, the same measurement can also be performed using a camera. The noise levels of a camera are slightly different, because camera pixels have limited well depth W, and per-pixel read noise n_{pix} is approximately constant regardless of integration time (this latter assumption is valid insofar as thermally-generated electron hole pairs are insignificant compared to read noise; this will occur if the pixel area is small, the integration time is short and the sensor temperature is low, all of which are valid assumptions for a modern camera sensor imaging calcium or voltage activity). Therefore many pixels must be summed together in order to reach the necessary photon counts; the total noise is consequently the sum of the noise from each pixel in quadrature. The number of pixels needed to capture a total of F photons is, of course, heavily dependent on the spatial distribution of the

fluorescence signal, but the lower bound is simply F/W, i.e. each pixel in the ROI captures the same number of photons. Consequently, a lower bound on n_{dark} can be given as:

$$n_{dark} = \sqrt{\frac{F}{W} n_{pix}^2}$$

Eq. 7

For a camera, the quadratic expression therefore simplifies to

$$\left(\frac{\Delta F_{\%}}{SNR}\right)^2 F^2 - F - \frac{F}{W} n_{pix}^2 = \left(\frac{\Delta F_{\%}}{SNR}\right)^2 F^2 - \left(1 + \frac{n_{pix}^2}{W}\right) F = 0$$

Eq. 8

Ignoring the trivial solution $F = 0$, the required number of captured photons for a camera F_{cam} is thus given by:

$$F_{cam} = \left(1 + \frac{n_{pix}^2}{W}\right) \left(\frac{SNR}{\Delta F_{\%}}\right)^2$$

Eq. 9

The same equation can be used for the analysis of a Single Photon Avalanche Diode (SPAD) array, which is a modern "camera" composed of an array of avalanche photodiodes. Being pixelated detectors with a limit to the number of detectable photons per pixel, pixels must be summed together to achieve the necessary dynamic range to measure small changes in the fluorescence signal.

It should be noted that F and F_{cam} refer to the number of detected photons, not the number of photons emitted by the sample. Comparing the necessary number of emitted photons requires dividing by the microscope's photon collection efficiency, the system transmission coefficient and the photon detection efficiency of the detector. Comparisons made in this way should be treated with care, as a system configuration optimal for one detector may not be optimal for another. For example, the numerical aperture of a tube lens will often be significantly lower for a camera compared to a point detector as there is no need to form an aberration-free image for a point detector; the resultant increase in photons captured by the tube lens can be quite significant in the case of scattering samples. Furthermore, scattered photons can be usefully captured by a single point detector whereas in the case of a camera the majority will impact the sensor outside of a small ROI and not be measured (or even worse, affect the signal in another ROI leading to crosstalk).

With all the above caveats in place, the data presented are simulated as follows: for each detector, the number of photons required to overcome the combination of detector noise and photon shot noise is calculated as detailed above. In the case of pixelated detectors (i.e. the camera and the SPAD array) the well depth W is reduced such that the probability of a pixel saturating is less than the required $\Delta F_{\%}$. This was done by calculating the cumulative distribution function of a Poisson function evaluated at the actual well depth W, with integer values of the λ parameter up to the value of W. The value of the λ parameter at which $1 - CDF < \Delta F_{\%}$ where CDF is the cumulative distribution function, was taken as the mean pixel value. Once the required number of photons was calculated, it was divided by the photon detection efficiency of each detector. A MATLAB script performing these calculations can be obtained from https://www.imperial.ac.uk/rowlands-lab/.

Table S1. Properties of selected detectors

Detector	Read noise	Maximum signal	Photon detection efficiency
**PMT: Hamamatsu H9305-03**	3720 photons / s [a]	6.24×10^8 photons / s, 10^5 gain [b]	30% [d]
**Photodiode: Femto LCA-S-400K-SI-FST**	2,680,000 photons / s	4.29×10^{12} photons / s [c]	83% [e]
**SiPM: Hamamatsu C13366-3050GA**	937,000 photons / s	12.6×10^9 photons / s	40%
**SPAD array: Pi Imaging SPAD512S**	25 photons / s / pixel	1×10^5 photons / s / pixel	50%
**Camera: Photometrics Kinetix**	2 photons / pixel / frame	200 photons / pixel / frame	96%

[a]Read noise calculated as dark current/ radiant sensitivity. [b]Maximum signal calculated as (max current/ charge of an electron)/ electron multiplication gain [c]Maximum signal calculated as max current/ charge of an electron. [d]Value for 555U photocathode from ***Photomultiplier Tubes : Basics and Applications*** 4th edition by Hamamatsu. [e]Value derived from peak 0.6A/W sensitivity at 900nm on datasheet; energy of a photon is Planck Constant × Speed of light in a vacuum / wavelength, so 1W of 900nm photons is 4.53×10^{18} photons per second. 0.6A / charge on an electron is 3.74×10^{18} electrons per second. 3.74 / 4.53 ≈ 83% or 0.83 electrons per incident photon.

S2. DMD projection speed

The number of ROIs that can be measured and the rate at which these measurements can be taken is controlled directly by the frame rate of the DMD. Custom software was written in LabView, and the frame exposure time for each mask on the DMD was manually adjusted within the software used to run the experiments. The minimum exposure time at which the DMD was used was 100 µs per frame (10 kHz frame rate) to avoid DMD firmware instability at higher rates, though it is possible to achieve a frame rate of up to 22 kHz. The inter-frame time (i.e. the time between the masks being displayed while the DMD was switching) was measured. At frame exposure times of 100 µs, 1000 µs, 10000 µs, and 100000 µs the inter-frame time between masks was 60µs. This time is included in the frame exposure times of the DMD. Over the course of a 10 second trial targeting 10 regions, and at a frame rate of 10 kHz, each region is being targeted at a 1 kHz rate and only 6% of the experimental time per sample is taken by the DMD switching between masks. This implies that fluorescence signals with a bandwidth of 500 Hz (after considering Nyquist sampling) can be recorded, which comfortably exceeds the bandwidth of most fluorescent indicators in biological samples [7].

S3. Optical Diagram for inverted setup

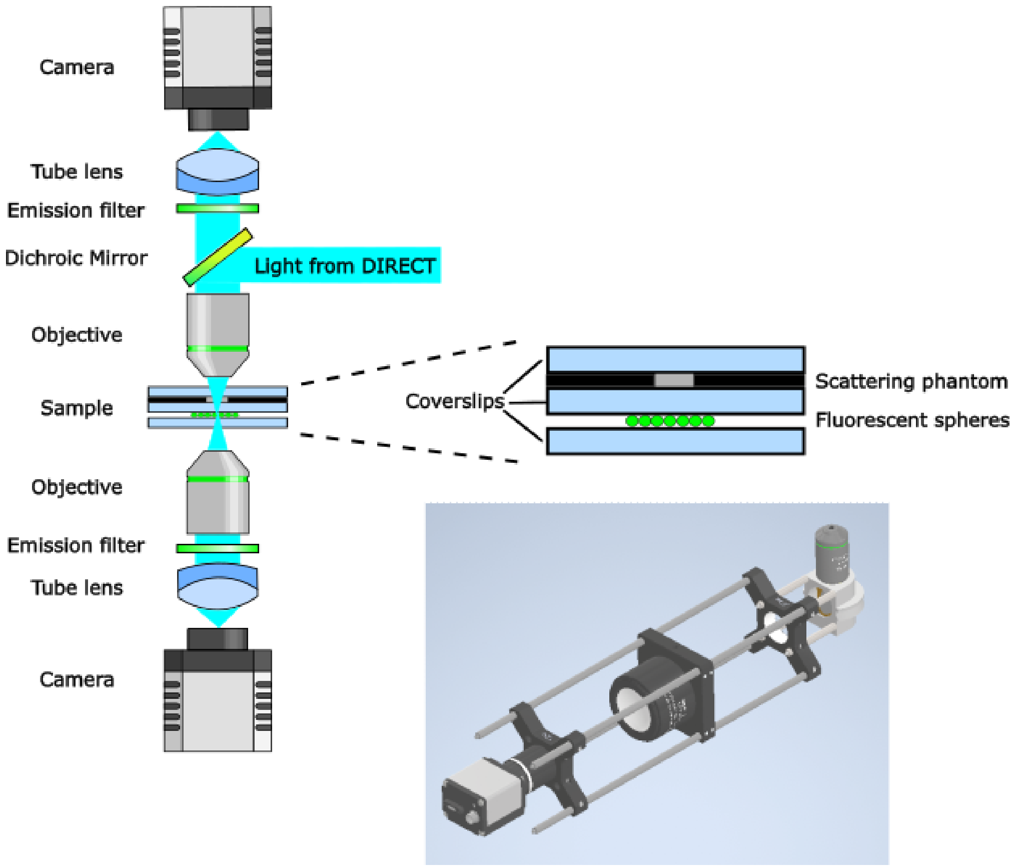

Fig. S3 Experimental design for scattering tolerance assessment. Left: upright and inverted microscopes. The inverted microscope is fitted onto the existing upright microscope at the location of the condenser lens using a custom milled objective holder with an Olympus condenser dovetail. Right: scattering sample design. From bottom up, coverslip, thin layer of fluorescent microspheres, coverslip, 200 µm thick spacer with 5mm diameter well containing Intralipid 20% phantom, and coverslip. Inset, CAD model for inverted microscope

S4. DIRECT vs widefield imaging

Before recording data at high speed it is illustrative to assess the effect of the targeted ROIs, using a camera rather than a single-point detector. ASAP3-expressing neurons across the field of view of the camera were targeted and a mask containing the selected neurons was generated. The resulting image of the projected mask was compared to a widefield image taken of the same FOV. The images were normalized such that the targeted neurons had the same average intensity and the background/non-targeted regions were compared (Fig. S4). In the widefield image, the target intensity was negligibly higher than the background intensity (target to background ratio = 0.9293) whereas the ratio of intensity of the targeted region compared to the non-targeted region of DIRECT was much larger (target to background ratio = 6.7440). While the intensity of the light inside the ROIs was similar, the intensity of the background of the widefield image was much greater than that of the ROI image. By using DIRECT, the background noise of the image was drastically reduced while the target intensity was unchanged. This allows for recordings of voltage activity with less noise while preserving other fluorescent neurons for further experimental recording.

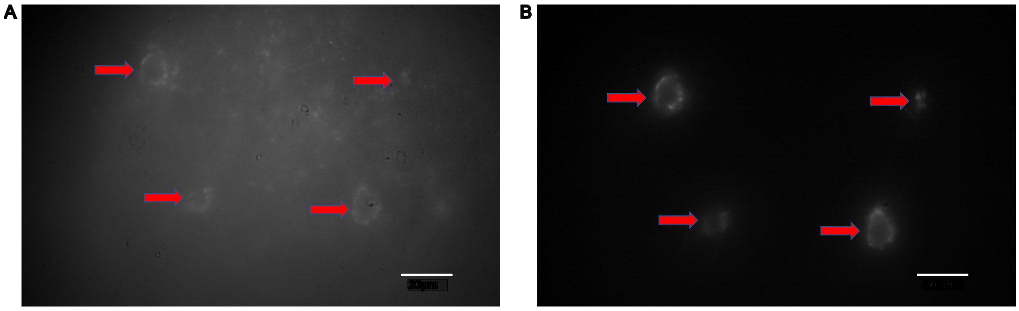

Fig. S4 Widefield vs DIRECT imaging**.** Representative example of widefield voltage imaging compared to DIRECTs targeted illumination. A. Widefield image of an *ex vivo* sample with GEVI containing neurons, red arrows on widefield example indicate neurons selected for targeting. B. The same *ex vivo* sample with targeted imaging of the selected neurons, demonstrating the removal of background fluorescence resulting from the use of targeted imaging. Scale bars = 20µm

S5. Methods

1.1 Optical design

The DIRECT system is as follows: light from a 488nm laser (Coherent Sapphire 488-200) passes through a 4.5× beam expander (Thorlabs A220TM-A and LBF254-050-A) and strikes a DMD (Vialux V-7001) located conjugate to the sample plane of the microscope. An alternative illumination path is also available, for light-emitting diode (LED) rather than laser illumination; after the beam expander but before the DMD, a mirror (Thorlabs MRA25-E02, placed in a removable cube: Thorlabs DFM1B-M and Thorlabs DFM1T2 cube insert) can be placed to switch to the LED.

The light reflected from the DMD passes through a 4f lens system (for characterization experiments: four Thorlabs LA1256-A lenses in Plössl pairs, for voltage imaging experiments: two Thorlabs LA1417-A lenses) onto an intermediate plane (where optionally a mask may be placed) and into a tube lens (Olympus SWTLU-C) before reflecting off a dichroic mirror (Semrock Di03-R488-t1-25x36 mounted in a Thorlabs DFM1/M removable filter cube, which is in turn mounted in a custom holder with Olympus dovetails) and through the objective (20x Olympus UPlanSApo for fluorescent bead experiments, either 16x Nikon CFI75 LWD 16X W or 60x Olympus LUMPLFLN60XW for voltage imaging experiments), onto the sample. The light emitted from the sample passes back through the dichroic and an emission filter (Semrock ff03-525/50-25), and is recorded by either a camera (Basler acA1920-155um or IDS U3-3080SE-M-GL) or PMT (Hamamatsu H9305-03). In all cases, signals from the PMT were digitized using an ADC module / oscilloscope (Pico Technology Picoscope 5444D).

DIRECT is intended to be inserted to the infinity path of a standard fluorescence microscope; in our demonstration we inserted it between the built-in fluorescence illuminator (Olympus BX-RFAA) and the trinocular head (Olympus U-TR30-2) of an Olympus BX51WI or BX61 using a custom-machined dichroic holder with Olympus dovetails. CAD models for all custom parts are available at https://www.imperial.ac.uk/rowlands-lab/; an optical diagram can be seen in Fig. 1A. The system was designed and optimized using Autodesk Inventor Professional 2023 and Zemax OpticStudio.
For characterization experiments a camera was used rather than a PMT as an image is required to assess spatial resolution. However, in its application, DIRECT has been designed to use a PMT to allow user to record through scattering media where a camera be unable to do so. Additionally, cameras have limited temporal resolution to perform experiments with rapid fluorescence fluctuations. The spatial resolution of a PMT cannot be measured as it is a single point detector and collects all photons emitted by the sample.

Table S6. Microscope configurations for different experiments

	Objective	Detector/Camera	Microscope
Characterization experiments	20x Olympus UPlanSApo	IDS U3-3080SE-M-GL	Olympus BX61
Neurophysiological recordings	16x Nikon CFI75 LWD 16X W or 60x Olympus LUMPLFLN60XW	Hamamatsu H9305-03 Basler acA1920-155um	Olympus BX51WI

1.2 Experimental control and ROI generation

The DMD allows arbitrary binary patterns to be projected onto the sample at high speed. This can be used to project individual ROIs. A custom software interface written in LabVIEW 2018 calling the functions from the Vialux ALP4.3 Dynamic Link Library (DLL) was used to upload binary sequences to the DMD; ROIs and patterns could be generated, and the rate of switching between each frame of the sequence could be selected for each experiment.
For an ROI projection experiment (where a sequence of ROIs was illuminated in quick succession), a widefield image was taken of the full FOV of the sample with no binning. The picture was loaded into the software interface and regions of interest were hand-drawn around the selected targets. Each region of interest was converted into a binary mask and uploaded to the DMD. To confirm accuracy of ROIs, all masks were combined into a single mask then projected onto the sample and a picture was captured.
To measure the light throughput of the system, representative ROIs were generated for a 16x and 60x magnifications. The average power of a ROI for a 60x objective was 0.4027mW and 0.0676mW for a 16x objective (n = 10 ROIs) at a starting laser power of 200mW.

1.3 Synchronization

Because the DMD could not be synchronized to the photodetector / ADC clock (or vice versa), synchronization between the two had to be performed offline. A start trigger was used to initialize the projection of the pattern

sequence, after which the DMD and ADC board then ran asynchronously. Because the DMD was reset between frames (i.e. all mirrors returned to an 'off' position) the signal on the detector dropped to zero every frame, before rapidly increasing to a nonzero value, acting as an embedded sample clock. The ADC sampled the signal much faster than the pattern switching frequency, and could therefore capture the rapid increase and decrease. A custom MATLAB script was used to recover the embedded sample clock (also compensating for slow drift between the DMD and ADC clocks); the script could optionally take an average of each 'on' signal for denoising and data reduction purposes.

1.4 Projection Speed

Masks of differing sizes were projected by the DMD at frame exposure times of 100 µs, 1000 µs, 10000 µs, and 100000 µs onto a fluorescent sample. Fluorescence was detected using a photodiode (Laser components Ltd Photoreceiver LCA-S-400K-SI) and oscilloscope (Picoscope 2204) sampling at 100 kS/s. Mask switching was detected by identifying troughs between changing voltage levels as described previously.

1.5 Photobleaching

To assess on- and off-target photobleaching, a sample consisting of a close-packed array of 10× diluted 100nm fluorescent microspheres (Fluoresbrite YG Carboxylate Microspheres 0.10µm) was placed in the field of view. A reference image was taken of the whole field of view with all pixels illuminated. ROIs consisting of three ~55 µm diameter circles were projected onto the sample in quick succession (10 kHz), with the sequence repeating continuously throughout the experiment. Camera frames were taken continuously (exposure time 15 ms, laser intensity at the sample 60 mW over a ~425×350 µm area) and the exposure continued for a period of 20 minutes. Finally a comparison image was taken of the whole field of view (once again with all pixels illuminated). The experiment was then repeated with the laser power increased by a factor of three (exposure time 15 ms) (accounting for the threefold reduction in exposure duration caused by the sequential ROI exposure durations). A final experiment was done with all pixels illuminated for the whole duration of the experiment (exposure time 15 ms) (simulating wide-field camera exposure). In each case a new, unexposed region of the sample was used.

1.6 Projection resolution and scattering tolerance

To assess the resolution of the targeting system under various imaging conditions, lines were projected by the DMD and imaged onto a camera (IDS U3-3080SE-M-GL) rather than a single point detector, in order to eliminate the effect of sample heterogeneity. Tests were performed by projecting two single-mirror-width lines onto a sample with rows of OFF mirrors between them. The sample was composed of a microscope slide with a thin layer of dried fluorescent microspheres (Fluoresbrite YG Carboxylate Microspheres 0.10 µm) and a coverslip. The average pixel intensity was taken horizontally across the vertically projected lines to determine the intensity profile and separation of the lines.

The resolution of the targeting was assessed in controlled scattering conditions. An inverted microscope (Figure S3) consisting of a microscope objective (Olympus UPlanApo 20x), elliptical mirror (Thorlabs BBE1-E02), tube lens (Thorlabs TTL200MP), emission filter (Chroma HQ535/50 x), and camera (IDS U3-3080SE-M-GL) was placed below the sample to acquire the transmission image of the pattern after passing through a scattering medium. The inverted microscope was fitted to the upright microscope setup at the location of the condenser. The condenser lens was removed from its mount and a custom milled dovetail-adaptor (CAD model available), holding the objective and elliptical mirror, was placed.

The above projection resolution experiment was repeated, this time through the scattering sample and both transmission and reflection images were captured. Scattering phantoms were created using dilutions of Intralipid 20% (Sigma-Aldrich 68890-65-3). Dilutions used were 0.1%, 0.5%, 1%, 2%, 4%, 8%, and 10%. The sample consisted of a thin layer of fluorescent microspheres (Fluoresbrite YG Carboxylate Microspheres 0.10 µm) between two coverslips, a 200 µm thick spacer with a 5 mm diameter well containing a scattering phantom and a third coverslip on top (Figure S3). The spacer was made using made 3M 9088 White Double Sided Plastic Tape; the well was made using a 5 mm hole punch.

1.7 Acute brain slice preparation

All experimental procedures were performed at Imperial College London UK in accordance with the United Kingdom Animal Scientific Procedures Act (1986), under Home Office Personal and Project Licences following appropriate ethical review.

Adult C57BL/6 mice were injected with 1µl of AAV9.mDlx-ASAP3-Kv into the somatosensory cortex. After 3 weeks expression time, mice were terminally anaesthetized with ketamine/xylazine, and transcardially perfused with ice-cold sucrose-based cutting solution (osmolarity of 300-310), with the composition (in mM): 3 KCl, 26

$NaHCO_3$, 1.25 NaH_2PO_4, 3 Na pyruvate, 0.5 $CaCl_2$, 4 $MgCl_2$, 190 sucrose, and 25 dextrose (pH7.4 bubbled with carbogen), and brain extracted. Coronal slices of 250µm thickness were cut with a Leica TS1200 vibratome and immediately transferred to holding artificial cerebrospinal fluid (ACSF, osmolarity of 300-310) at 34°C, with the composition (in mM): 126 NaCl, 3.5 KCl, 26 $NaHCO_3$, 1.25 NaH_2PO_4, 2 $CaCl_2$, 2 $MgSO_4$ and 10 dextrose (pH7.4 bubbled with carbogen), and allowed to recover for 30 min before transferring to room temperature. Recording ACSF (osmolarity of 300-310) was heated to 34°C and similar to holding ACSF in composition except with 1.2 $CaCl_2$ and 1 $MgSO_4$.

1.8 Neurophysiological activity recordings

DIRECT vs Widefield To assess the functional use of DIRECT for biological applications in scattering tissue, the spatial resolution of DIRECT was compared with widefield imaging in an *ex vivo* acute mouse brain slice preparation with neurons expressing the voltage indicator soma-targeted ASAP3. Previous papers have also shown the reduction of background light using a DMD to target neurons [17], and this was also confirmed with DIRECT. A mask of four neurons was projected by the DMD and a widefield image of the same FOV was also captured onto a camera (Basler acA1920-155um).

Neurophysiological Activity A widefield image was captured using the laser at 20 mW and ROIs drawn around the targeted neurons as described in previous section. During the experiment, the laser power was set to 200 mW. Multi-ROI PMT recordings with extracellular stimulation: an extracellular stimulation electrode was placed in close proximity to the targeted neurons. Individual ROI masks of each target were loaded into the DMD, the experiment was triggered to begin. A single pulse was given by the electrode 0.5 seconds after the experiment began. It was followed by five consecutive pulses at 20 Hz. The pulse current was 10-100 µA with 200 µs pulse duration. The DMD switched between all ROI masks in a continuous pattern with a mask exposure time of 100 µs. Emitted photons were recorded by a PMT with an oscilloscope (PicoScope 5444D PC Oscilloscope 200 MHz 4 channel, Pico Technology) sampling at 1 MHz for two seconds.

1.9 Data analysis

All system characterization, image, and neurophysiological data analysis was performed in Matlab R2022a.

System Characterization

Projection speed. Multi-ROI Picoscope data was converted to Matlab files, the troughs between ROIs were identified and the average time of the troughs was taken.

Photobleaching assessment. For ROI images, the mask used to project the ROIs was used to isolate the target regions of the sample, and the average value of targeted regions was taken. For full FOV images, the average value of each image gathered in the time series was taken for full FOV images. The average values of each image were normalized to the starting image value. Photobleaching was assumed to be monoexponential with an offset to account for camera pixel offsets. The time series means were therefore curve-fitted using a nonlinear least square method to the following equation:

$$f(x) = ae^{(-bx)} + c$$

Eq. 10

where *a*, *b* and *c* are fitted constants. For off target results, the non-targeted regions were isolated in the full FOV before and after images; the average was taken and normalized to the starting value. The change was calculated by subtracting the after value from the before value.

Resolution and scattering tolerance. Images were loaded, normalized, and lines were manually isolated. The average pixel intensity was calculated across rows of the image. FWHM was measured by calculating the half maximum and interpolating the width at the value. Theoretical mirror widths were calculated for a diffraction limited system. To model the imaging of the projected DMD patterns under diffraction-limited conditions, the imaging pipeline was simulated using a Fourier optics approach. A simulated point spread function was constructed assuming a diffraction-limited optical system along with a grid of pixels based on the detector and DMD pixel widths. This pixel grid was sufficiently subsampled to facilitate the inclusion of intra-mirror gaps of the given DMD fill factor. Columns of pixels in this grid, representing columns of DMD mirrors, were subsequently 'illuminated' thereby generating the projected DMD pattern. This projected pattern was then convolved with the PSF to simulate the final diffraction-limited image of the DMD pattern.

Neurophysiological Activity

Individual traces of neurophysiological data recorded onto the PMT were separated using a lab-built function. The function fits a square wave (with variable period and duty cycle) to the data to separate each ROI activity trace. The data from each segment separated by the square wave is averaged to a single data point and the points are placed into a vector for each ROI. Data is presented in raw format, or with a moving average smoothing filter

with spans of 10 data points for multi-neuron PMT recordings. Action potentials for $\Delta F/F$ measurements were calculated by taking the $\Delta F/F$ of each value in a subset of the data, known to contain the first action potential and selecting the maximum resulting value. The SNR was calculated by taking the maximum $\Delta F/F$ and dividing by the standard deviation of the baseline signal.
